# Nutritional Management for Preterm Infants with Common Comorbidities: A Narrative Review

**DOI:** 10.3390/nu17121959

**Published:** 2025-06-09

**Authors:** Cheng-Yen Chen, Mei-Yin Lai, Cheng-Han Lee, Ming-Chou Chiang

**Affiliations:** 1 Division of Neonatology, Department of Pediatrics, Chang Gung Memorial Hospital and Chang Gung University College of Medicine, Taoyuan 33305, Taiwan; as14532000@gmail.com (C.-Y.C.); lmi818@msn.com (M.-Y.L.); 2 Graduate Institute of Clinical Medical Sciences, Chang Gung University College of Medicine, Taoyuan 33305, Taiwan; 3 Division of Neonatology, Department of Pediatrics, Changhua Christian Children’s Hospital, Changhua 50006, Taiwan; 129130@cch.org.tw; 4 Division of Respiratory Therapy, Chang Gung Memorial Hospital, Taoyuan 33305, Taiwan

**Keywords:** enteral nutrition, extremely low birth weight infant, morbidities, nutrition, parenteral nutrition, preterm neonates, very low birth weight infant

## Abstract

The complications observed in preterm infants are largely attributable to underdeveloped organ systems and inadequate nutritional stores at birth. Insufficient nutritional support can further exacerbate persistent sequelae, such as bronchopulmonary dysplasia (BPD), metabolic bone disease of prematurity (MBDP), and retinopathy of prematurity (ROP). As a result, clinicians have collaborated to develop optimal nutrition strategies for preterm neonates. However, these clinical nutrition plans may be hindered by several factors, including fluid restrictions due to patent ductus arteriosus (PDA) and delayed enteral nutrition following necrotizing enterocolitis (NEC). Modified strategies for specific conditions can help prevent further deterioration, but inadequate nutritional support may limit organ growth and contribute to additional complications. Achieving an optimal balance between nutritional support and managing specific medical conditions varies across institutions. In addition to fluid balance and energy intake, supplementary nutrition—such as vitamins and probiotics—plays a crucial role in disease prevention. Drawing on recent evidence and our clinical experiences with neonatal nutritional strategies, this review article summarizes the specialized nutritional management required for preterm neonates with conditions such as BPD, NEC, MBDP, PDA, and ROP.

## 1. Introduction

Nutritional management has been an essential aspect of neonatal care for preterm infants, particularly due to its impact on neurodevelopmental outcomes [1]. Because of their immature organ development and limited nutritional reserves, preterm infants require more aggressive and delicate nutritional support than term infants to achieve healthy catch-up growth [2]. The European Society for Pediatric Gastroenterology, Hepatology, and Nutrition (ESPGHAN), the American Academy of Pediatrics (AAP), the American Society for Parenteral and Enteral Nutrition (ASPEN), and various other organizations have focused on reviewing the literature related to this topic and have proposed different nutritional recommendations to meet the physiological needs of preterm infants during their growth [3,4,5,6,7,8,9,10,11,12,13,14,15]. Nutritional recommendations for preterm infants have been widely studied; however, comorbidities are common and often influence nutritional tolerance and requirements in early life. Comorbidities often seen in preterm infants, such as bronchopulmonary dysplasia (BPD), metabolic bone disease of prematurity (MBDP), and retinopathy of prematurity (ROP), pose serious health challenges that contribute to poor postnatal growth and economic burdens [15,16,17]. Necrotizing enterocolitis (NEC) is a significant bowel complication related to postnatal growth issues, arising from delayed enteral feeding and extended parenteral nutrition (PN) [16]. The observed association between the management of hemodynamically significant patent ductus arteriosus (hsPDA) and impaired postnatal growth highlights the importance of implementing condition-specific nutritional strategies for preterm infants affected by hsPDA [18]. Despite the growing volume of literature and international nutrition guidelines, there is still a lack of a comprehensive review that consolidates current evidence and translates it into condition-specific nutritional strategies. Numerous researchers have committed themselves to investigating optimal nutritional management for these conditions while minimizing cost and risk. Therefore, this narrative review aims to summarize and interpret the most relevant recent studies, expert consensus statements, and international guidelines to assist clinicians in modifying nutritional plans according to the presence of common neonatal comorbidities. This approach intends to bridge the gap between general recommendations and the real-world complexity of neonatal nutrition in high-risk infants.

## 2. Methods

This article is structured as a narrative review. We conducted a targeted literature search of PubMed, Scopus, and Cochrane Library databases using combinations of the following keywords: “preterm infants”, “nutrition”, “feeding”, and each of the comorbidities discussed (e.g., “bronchopulmonary dysplasia”, “necrotizing enterocolitis”, “retinopathy of prematurity”, etc.). The search included English-language articles published between January 2010 and February 2024.

Inclusion criteria were (1) studies focusing on nutritional interventions in preterm infants with specified comorbidities, (2) randomized controlled trials, cohort studies, and meta-analyses, and (3) relevant international clinical practice guidelines (e.g., ESPGHAN, AAP). Exclusion criteria included duplicate reports, studies focusing solely on term infants, or those without nutrition-related outcomes.

To assess the strength and reliability of evidence cited for specific interventions, we applied the GRADE (Grading of Recommendations Assessment, Development and Evaluation) methodology. Each included study was independently assessed by three certified neonatologists with clinical and academic expertise in neonatal nutrition, based on five GRADE domains: risk of bias, inconsistency, indirectness, imprecision, and publication bias. The quality of evidence for each outcome was classified as high, moderate, low, or very low. In addition, we evaluated the strength of recommendations derived from the evidence, which were graded on a scale from weak to strong, depending on the balance between desirable and undesirable consequences, overall quality of evidence, and clinical applicability. Any discrepancies in the assessments were resolved through consensus discussions among the reviewers. If consensus could not be reached, the final rating was determined by majority agreement. The review process, including study selection and data extraction, was independently conducted by the authors. Findings were synthesized and presented in structured, comorbidity-specific nutritional recommendations.

## 3. Bronchopulmonary Dysplasia (BPD) and Nutritional Challenges

**Figure 1:**
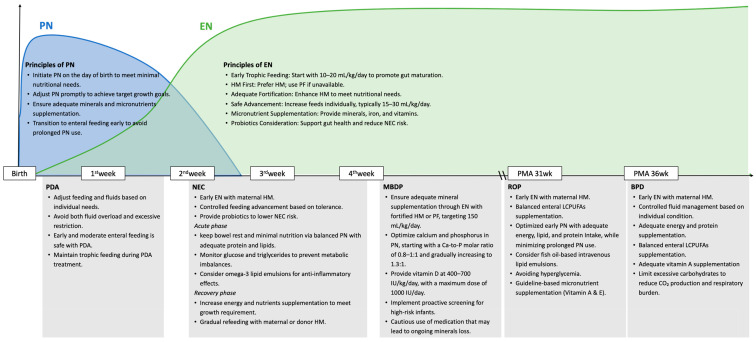
Principles of Nutritional Management for Preterm Infants and Key Focus Areas for Managing Comorbidities during the Postnatal Period. The minimal nutritional needs on the day of birth are as follows: Energy, 50–60 kcal/kg/day; glucose, 6–12 mg/kg/day; amino acids (A.A.), ≥1.5 g/kg/day; and lipids, 1.5 g/kg/day. For target growth, the nutritional goals are energy 100–120 kcal/kg/day, glucose 12–14 mg/kg/day, amino acids 2.5–3.5 g/kg/day, and lipids 3.5–4.0 g/kg/day, which should be achieved as soon as possible. Abbreviations: PN: parenteral nutrition; EN, enteral nutrition; HM, human milk; NEC, necrotizing enterocolitis; PMA, postmenstrual age; PDA, patent ductus arteriosus; MBDP, metabolic bone disease of prematurity; ROP, retinopathy of prematurity; LCPUFAs, long-chain polyunsaturated fatty acids; BPD, bronchopulmonary dysplasia.

Bronchopulmonary dysplasia is a chronic lung disease that impacts preterm neonates who require extended mechanical ventilation and oxygen therapy. Advances in neonatal care have improved survival rates, leading to a higher incidence of BPD. As lung growth continues throughout the neonatal period, nutritional support is crucial for fostering lung development and preventing BPD. Recent studies on nutrition strategies for managing BPD are reviewed and summarized in Figure 1.

### 3.1. Calories and Fluid Intake

According to ESPGHAN guidelines, premature neonates require at least 45–55 kcal/kg/day at birth, with this amount increasing to 90–120 kcal/kg/day [8]. However, infants with BPD exhibit higher resting energy expenditure (REE) due to increased breathing effort. Lima et al. reported a 20–30% increase in REE in preterm infants with BPD [19]. Additionally, several studies have shown that inadequate calorie intake in the first two weeks correlates with an increased risk of BPD [20,21,22,23]. To mitigate this, we strongly recommend that energy intake for preterm infants begin at 50–60 kcal/kg/day and gradually advance to 130–150 kcal/kg/day to support optimal postnatal growth. (Strength: Strong; Quality of evidence: Moderate).

Fluid management poses a challenge, as excessive fluid intake has been linked to poor respiratory outcomes. Milanesi et al. reported a negative correlation between fluid volume at 3–4 weeks of life and the risk of BPD (Quality of evidence: Very Low) [20]. Matsushita et al. found that severe fluid overload (>15% of body weight within 72 h) increased mortality and prolonged mechanical ventilation (Quality of evidence: Low) [24]. Clinicians should modify fluid management based on daily fluid balance, urine output, weight, and electrolyte levels to optimize outcomes. While fluid restriction can help reduce pulmonary edema, it must be balanced with adequate nutritional intake to prevent growth failure and metabolic imbalances.

### 3.2. Enteral Nutrition (EN) and Human Milk

Early enteral feeding has been associated with a lower risk of BPD. Delayed initiation of enteral feeding often leads to prolonged PN, which is linked to late-onset sepsis and an increased risk of BPD [25]. Milanesi et al. found that extended PN was associated with a higher incidence of BPD (Quality of evidence: Low) [20]. Lin et al. reported that a 10% increase in the enteral feeding-to-total fluid intake ratio during the second week of life reduced BPD risk by 55.6% (Quality of evidence: Low) [26]. These protective effects may be attributed to gut microbiota modulation and metabolic benefits.

Human milk provides protective effects due to its bioactive components, which possess antioxidant and immune-modulating properties [27,28]. Xu et al. found that feeding ≥50 mL/kg/day of human milk during the first four weeks significantly reduced the risk of BPD [29]. Early enteral feeding with human milk is recommended once neonates are stabilized, with gradual increases in feeding volume as tolerated (Quality of evidence: Moderate). In the absence of maternal milk, donor human milk serves as an acceptable alternative; however, it often requires fortification to adequately meet the elevated nutritional requirements of preterm infants (Strength: Strong; Quality of evidence: Moderate).

### 3.3. Macronutrients: Protein, Carbohydrate, and Lipid

Protein: There is no direct link between high protein intake and the risk of BPD. Milanesi et al. found that preterm neonates receiving a calorie/protein ratio of <30 kcal/g had a higher incidence of BPD (Quality of evidence: Low) [20]. ESPGHAN guidelines recommend an initial protein intake of 1.5 g/kg/day, increasing to 3.5 g/kg/day [5]. Proper protein intake supports lung tissue repair and alveolarization, which are critical for managing BPD.

Carbohydrates: Carbohydrates serve as a major energy source for preterm neonates. Studies have indicated that lower carbohydrate intake in the first two weeks is associated with a higher risk of BPD due to inadequate energy supply [21,24]. A glucose infusion rate of 4–8 mg/kg/min, advancing to 10–12 mg/kg/min, is suggested to balance energy needs while avoiding excessive CO_2_ production [7]. High carbohydrate intake should be carefully monitored to prevent hyperglycemia, which may contribute to oxidative stress and inflammation.

Lipid: Lipids provide a high-calorie density with minimal fluid volume, making them ideal for preterm infants under fluid restrictions. Several studies have demonstrated an association between low lipid intake and increased risk of BPD [20,21]. Long-chain polyunsaturated fatty acids (LCPUFAs), particularly docosahexaenoic acid (DHA), have anti-inflammatory properties [30]. However, recent trials have shown that additional DHA supplementation does not significantly reduce the incidence of BPD (Quality of evidence: Moderate) [31,32]. ESPGHAN guidelines recommend initiating lipid emulsions once PN begins, with an upper limit of 4 g/kg/day to prevent proinflammatory effects [9]. Ensuring an appropriate balance of omega-3 and omega-6 fatty acids may further support lung development and immune function.

### 3.4. Vitamin A and Other Micronutrients

Vitamin A plays a crucial role in lung development. The reanalysis of the National Institutes of Child Health and Human Development Neonatal Research Network Vitamin A trial by Rysavy et al. found that intramuscular vitamin A supplementation reduced the risk of BPD (Quality of evidence: Moderate) [33]. However, concerns regarding pain and infection have limited its routine use. Rakshasbhuvankar et al. reported that high-dose enteral vitamin A (5000 IU/day) did not significantly reduce the severity of BPD (Quality of evidence: Moderate) [34]. Similarly, Meyer et al. found that early postnatal high-dose enteral vitamin A supplementation (5000 IU/kg/day for 28 days) did not lower the risk of moderate to severe BPD compared to a placebo group receiving 1000 IU/kg/day (Quality of evidence: Moderate) [35]. Due to the inconclusive benefits of additional vitamin A supplementation, ESPGHAN recommends a daily intake of 700–1500 IU/kg/day [36].

Other micronutrients, such as vitamin D, zinc, and selenium, also play essential roles in neonatal lung development and immune function. Studies have suggested that vitamin D deficiency is common in preterm infants and may contribute to increased inflammation and impaired lung maturation [37]. Ensuring adequate vitamin D levels through supplementation may support overall respiratory health. Zinc is another key nutrient involved in lung tissue repair and immune regulation, and its deficiency has been associated with prolonged mechanical ventilation in preterm neonates.

### 3.5. Summary

Ensuring adequate calorie intake, fluid balance, early enteral feeding with human milk, and appropriate macronutrient and vitamin A supplementation can improve respiratory outcomes in preterm neonates. In addition to macronutrients, attention should be given to essential micronutrients like vitamin D and zinc, which may further enhance lung development and immune resilience. A multidisciplinary approach involving neonatologists, dietitians, and respiratory therapists is essential for optimizing nutritional strategies in preterm infants at risk of BPD (Strength: Strong; Quality of evidence: Low to Moderate).

## 4. Necrotizing Enterocolitis (NEC), Critical Illness, and Nutritional Challenges

Necrotizing enterocolitis is a serious gastrointestinal disease affecting preterm newborns, often occurring around 3–4 weeks postpartum after the initiation of enteral feeding, especially in infants born before 32 weeks of gestation [38]. NEC is caused by several factors, including intestinal immaturity, dysbiosis, and inflammation of the bowel [39,40]. Other risk factors include formula feeding, perinatal hypoxia, and hemodynamic instability. The exact pathophysiology remains unclear, but inflammation and compromised mucosal defenses are key factors. Preventive nutrition strategies and supportive care of NEC are reviewed and summarized in Figure 1.

### 4.1. Preventive Strategies—Feeding Approach

Enteral feeding is essential for neonatal growth, reducing the duration of PN and associated complications [41]. However, the optimal time of initiation and progress of feeding remains controversial. A systematic review by Morgan et al. found that delaying enteral feeding for more than four days did not reduce the risk of NEC [42]. Rapid feeding increases NEC risk but does not significantly reduce the duration of complete enteral feeding [43], while Montealegre-Pomar et al. demonstrated that early feeding at 30–40 mL/kg/day accelerated complete enteral feeding without increasing NEC risk [44]. A practical guideline recommends initiating nutritional feeding at a rate of 10–20 mL/kg/day, with incremental increases of 15–30 mL/kg/day after 3–5 days, adjusted for clinical tolerance (Strength: Strong, Quality of evidence: Moderate) [15]. Feeding regimens in the neonatal intensive care unit (NICU) should be standardized to ensure consistency and minimize the risk of NEC [42]. Close monitoring of feeding intolerance, including bloating and bloody stools, is important for early detection of NEC.

### 4.2. Human Milk and Fortification

Human milk significantly reduces the occurrence of NEC. Lapidaire et al. reported that each 10% increase in human milk intake reduced the risk of NEC by 12% (Quality of evidence: Low) [45]. Human milk contains immunomodulatory factors that mitigate inflammation and support beneficial microbiota [46]. Human milk oligosaccharides (HMOs) function as prebiotics for Bifidobacterium species, reducing the development of NEC and sepsis [47,48]. Additionally, maternal milk is rich in anti-inflammatory cytokines and growth factors that aid intestinal development. Fortifying human milk with additional proteins and lipids enhances growth and reduces complications in extremely low birth weight (ELBW) infants [49].

### 4.3. Probiotics and Gut Microbiota

Gut microbiota composition significantly influences the risk of NEC [39,40]. Early establishment of beneficial bacteria inhibits pathogenic colonization [50]. Multiple studies support probiotic efficacy in the prevention of NEC [51]. ESPGHAN recommends Lactobacillus rhamnosus GG ATCC53103 or a combination of Bifidobacterium infantis Bb-02, Bifidobacterium lactis Bb-12, and Streptococcus thermophilus TH-4 for the prevention of NEC [52]. Probiotics should be introduced with EN if tolerated. However, cases of probiotic-associated bacteremia highlight the need for caution [53]. Combining probiotics with prebiotics (synbiotics) may enhance gut barrier function and reduce inflammation [54]. Current research explores the role of postbiotics, which may provide similar benefits without the risk of live bacterial translocation.

### 4.4. Supportive Care—Parenteral Nutrition (PN) and Electrolyte Balance

Management of NEC includes bowel rest, PN, and broad-spectrum antibiotics [55]. Monitoring electrolytes, liver and renal function, and blood gases can guide fluid and nutritional modifications [41]. Protein intake of 3.5–4.0 g/kg/day helps in intestinal repair [15]. Lipid intake of approximately 3 g/kg/day ensures an adequate calorie supply [15]. Prolonged PN may lead to intestinal failure-associated liver disease, which can be managed with lipid emulsions based on fish oil [56,57]. Trace minerals such as zinc and selenium play a vital role in intestinal healing and immune function [55]. Recent studies suggest that PN solutions based on amino acids may help reduce inflammation [55]. Careful weaning from PN to enteral feeds is necessary to promote intestinal adaptation and prevent feeding intolerance.

### 4.5. Nutritional Considerations in Critically Ill Infants

The metabolic demands of NEC-associated sepsis or post-surgical recovery transition from low to high REE [58]. Inflammation triggers catabolic responses, including glycogenolysis and protein degradation [59]. Overfeeding in the acute phase may lead to metabolic imbalance [60]. The optimal timing of PN remains controversial [61,62]. Regular monitoring of blood glucose can prevent hyperglycemia, and it is recommended to keep blood glucose levels below 180 mg/dL [63]. ESPGHAN recommends a minimum intake of 55–58 kcal/kg/day and 1.3–1.5 g/kg/day of protein to achieve a positive nitrogen balance [64]. Intravenous lipid supplementation may mitigate excess CO_2_ production but must be monitored for hypertriglyceridemia [9]. Omega-3 fatty acids may have anti-inflammatory properties that may be beneficial for the recovery of NEC [60]. Vitamin D supplementation has also been explored to support immune function and intestinal integrity [64]. The gut-lung axis is an emerging area of research highlighting the potential impact of EN on systemic inflammation and respiratory outcomes.

### 4.6. Early Enteral Feeding Resumption

Resuming enteral feeding after NEC remains challenging due to concerns over bowel inflammation and recurrent NEC. However, delayed feeding disrupts gut microbiota, promoting opportunistic pathogen overgrowth [40]. Studies suggest early enteral feeding in stable infants post-NEC reduces risks of sepsis and intestinal failure (Quality of evidence: Very Low) [65,66]. A meta-analysis study found earlier enteral feeding reduced recurrent NEC and post-NEC strictures [67]. Clinical guidelines recommend bowel rest for 3–7 days, then reintroducing enteral feeding at 10–20 mL/kg/day if the infant is stable [15]. Human milk remains the preferred choice. If feeding intolerance arises without the recurrence of NEC, adjusting feeding volume instead of discontinuation is advisable. Short bowel syndrome, a severe NEC complication, requires individualized nutritional management [15]. Prebiotics combined with EN may help improve gut adaptation in NEC-recovering infants [54]. Some NICUs use small-volume continuous enteral feeding to enhance gut adaptation [61]. Emerging studies suggest that intestinal rehabilitation programs combining nutritional, pharmacologic, and surgical approaches may improve long-term outcomes.

### 4.7. Summary

Early enteral feeding, human milk, probiotics, and a carefully planned PN regimen can reduce the risk of NEC and improve neonatal outcomes. Individualized nutritional approaches optimize recovery and promote long-term gastrointestinal health in preterm neonates. Further research into advanced nutritional therapies—such as targeted lipid emulsions; prebiotic supplementation; and specialized amino acid formulations—may enhance the management of NEC. The integration of personalized nutrition strategies, based on biomarker analysis and gut microbiome profiling, could further refine NEC prevention and treatment.

## 5. Metabolic Bone Disease of Prematurity (MBDP) and Nutritional Challenges

Metabolic bone disease of prematurity, also known as osteopenia of prematurity, is a common nutritional and growth challenge in preterm infants. Due to inadequate bone mineral deposition during fetal development, the risk of MBDP increases with lower gestational age. The incidence is about 23% in very low birth weight (VLBW) preterm infants and 55% in those weighing <1000 g at birth [68,69]. MBDP can lead to growth retardation, osteoporosis, and even fractures. Approximately 80% of fetal bone mineralization occurs in the third trimester (32–36 weeks of gestation) [70]. Preterm birth disrupts this critical process, significantly increasing the risk of osteoporosis. Postnatally, immature organ function, clinical instability, medication use, and inadequate calcium and phosphorus intake further exacerbate bone loss and mineral deficiencies [71,72,73]. Early identification of high-risk infants and implementing proper screening, assessment, and nutritional strategies are key to preventing MBDP (Figure 1).

### 5.1. Screening

MBDP often has obscure early manifestations and only presents severe complications at a later stage. Therefore, regular monitoring of clinical indicators is crucial for early diagnosis. While serum calcium (Ca) and phosphorus (P) are commonly assessed, they are unreliable screening tools—Ca levels may remain normal despite bone calcium loss; and P levels can be influenced by parathyroid hormone (PTH). Alkaline phosphatase (ALP), a bone turnover marker, is considered a more reliable indicator of bone mineralization [74]. However, there is no universally accepted diagnostic criteria for MBDP. The AAP Committee guidelines recommend ALP and phosphorus screening for preterm infants <1500 g at 4–6 weeks after birth [3]. MBDP is influenced by prenatal factors like placental insufficiency, preeclampsia, intrauterine growth restriction (IUGR), VLBW, chorioamnionitis, and maternal vitamin D deficiency, which impair fetal bone accretion. Postnatal risks include feeding intolerance, prolonged PN, vitamin D deficiency, certain medications, sepsis, acidosis, organ dysfunction (renal, hepatic, or gastrointestinal diseases), and physical immobility, all contributing to impaired bone mineralization [75]. For at-risk infants, biochemical tests should be monitored every two weeks until they achieve full EN and maintain normal ALP and phosphorus levels [3]. Additional diagnostic tools include serum PTH, osteocalcin, urine Ca and P concentrations, and imaging methods such as quantitative ultrasound, X-ray, and dual-energy X-ray absorptiometry (DEXA) [76,77,78]. These assessments help in the early detection and management of MBDP, preventing severe complications.

### 5.2. Prevention

Early identification of high-risk MBDP cases, along with appropriate nutritional strategies and adequate calcium and phosphorus supplementation, is crucial for prevention. Although vitamin D intake is essential, it is generally not considered the primary cause of MBDP, especially in the first month of life when intestinal calcium absorption relies on passive transport rather than vitamin D-regulated active transport [79,80]. Insufficient calcium and phosphorus supplementation is the main pathogenic mechanism leading to MBDP. Maintaining calcium and phosphorus homeostasis is essential, and early establishment of EN with adequate supplementation is the most effective preventive strategy. Additionally, clinicians should carefully monitor medications that may increase the risk of MBDP, including loop diuretics and glucocorticoids.

### 5.3. Enteral Nutrition (EN)

Both the AAP [3] and ESPGHAN [4] provide EN recommendations on calcium, phosphorus, and vitamin D intake for VLBW preterm infants. The AAP recommends calcium 150–220 mg/kg/day, phosphorus 75–140 mg/kg/day, and vitamin D 200–400 IU/day, increasing vitamin D to 400 IU/day once the infant exceeds 1500 g with stable enteral feeding. ESPGHAN suggests a higher vitamin D intake (400–700 IU/kg/day, maximum dose 1000 IU/day), with calcium 120–200 mg/kg/day and phosphorus 70–115 mg/kg/day, maintaining an optimal Ca-to-P ratio of 1.5–1.7 mg/mg (≤1.8 in mass ratio), similar to human milk [79,81]. To meet these nutritional needs, VLBW infants should receive fortified human milk (24 kcal/oz) or preterm formula as their primary enteral nutrition source. Once feeding reaches 150 mL/kg/day, the intake of Ca, P, and vitamin D generally meets clinical recommendations [79].

### 5.4. Parenteral Nutrition (PN)

The latest ASPEN guidelines recommend providing daily calcium at 75 mg/kg/day and phosphorus at 45 mg/kg/day, with a Ca-to-P ratio of 1.7:1 (mg/mg) or 1.3:1 (mmol/mmol), in short-term PN to promote optimal bone strength in preterm infants [11]. However, studies indicate that a Ca-to-P molar ratio of 1.3–1.5:1 during the first postnatal week is associated with an increased risk of hypercalcemia and hypophosphatemia (Quality of evidence: Low) [82]. Therefore, the ESPGHAN guidelines recommend initiating PN with lower calcium (32–80 mg/kg/day or 0.8–2.0 mmol/kg/day) and phosphorus (31–62 mg/kg/day or 1.0–2.0 mmol/kg/day), maintaining a Ca-to-P molar ratio of 0.8–1:1 in the first days of life. This should then be gradually increased to calcium 100–140 mg/kg/day (1.6–3.5 mmol/kg/day) and phosphorus 77–108 mg/kg/day (1.6–3.5 mmol/kg/day), with a Ca-to-P molar ratio of 1.3:1, to optimize bone mineralization in growing preterm infants [6]. Achieving intrauterine mineral accretion through PN is challenging due to solubility limitations affected by factors such as amino acid composition, dextrose concentration, formulation pH, and temperature [83]. Additionally, calcium-phosphorus precipitation is a concern when increasing Ca and P concentrations in PN. Using glycerophosphate-containing PN solutions allows for higher Ca and P delivery while minimizing precipitation risk.

### 5.5. Treatment

For preterm infants with osteopenia, it is crucial to reassess whether enteral and parenteral nutrition meet recommended standards. If MBDP persists despite adequate calcium, phosphorus, and vitamin D intake, additional enteral or parenteral mineral supplementation may be considered. The initial supplementation dose is 20 mg/kg/day of calcium and 10–20 mg/kg/day of phosphorus, which can be gradually increased to a maximum of 70–80 mg/kg/day for calcium and 40–50 mg/kg/day for phosphorus [3]. Dosage adjustments should be guided by biweekly monitoring of biomarkers, including Ca, P, ALP, and PTH. For infants with vitamin D deficiency (i.e., serum vitamin D < 20 ng/mL or <50 nmol/L), high-dose vitamin D supplementation is reasonable [84]. However, while this approach reduces vitamin D deficiency, there is no clear evidence that it improves MBDP or bone mineralization (Quality of evidence: Moderate) [85]. Additionally, active vitamin D analogs such as alfacalcidol and calcitriol are indicated for renal or hepatic dysfunction or vitamin D activation defects but may not provide additional benefits for MBDP treatment [84].

### 5.6. Summary

Early identification and maintaining calcium-phosphorus homeostasis are key to preventing MBDP. Insufficient calcium and phosphorus intake is the main cause, while vitamin D plays a supportive role. Fortified human milk or preterm formula is essential to meet mineral requirements. A Ca-to-P molar ratio of 1.3:1 in PN supports bone mineralization. Implementing proper nutritional strategies, including careful medication monitoring, is vital for preventing MBDP in preterm infants.

## 6. Patent Ductus Arteriosus (PDA) and Nutritional Challenges

PDA alters the hemodynamic status of preterm infants by diverting systemic blood flow into the pulmonary circulation (left-to-right shunting), leading to pulmonary over-circulation, pulmonary edema, pulmonary hemorrhage, or left heart failure. The ductal steal effect reduces systemic perfusion, compromising organ function. Particularly, impaired enteral perfusion may contribute to feeding intolerance and increase the risk of NEC and neonatal mortality [86,87]. Management of hsPDA often involves nonselective cyclooxygenase inhibitors like indomethacin and ibuprofen. However, these agents induce visceral vasoconstriction, which—when combined with the ductal steal effect—may further exacerbate bowel ischemia; increasing the risk of spontaneous intestinal perforation (SIP) and NEC [88,89]. Balancing EN while minimizing PDA-related complications remains a key challenge for clinicians. Nutritional strategies for preterm neonates with PDA are summarized in Figure 1.

### 6.1. Fluid Management in PDA

Fluid restriction is commonly used to reduce left-to-right ductal shunting and prevent pulmonary fluid overload in PDA. A study by Stephens et al. found that high fluid intake (>170 mL/kg/day) in the first days of life increased PDA risk in ELBW infants (Quality of evidence: Low) [90]. Additionally, fluid overload in the first week not only raised the incidence of hsPDA but also prolonged its persistence [91,92]. However, fluid restriction often results in reduced energy and protein intake, potentially impairing postnatal growth [93]. A recent systematic review by MacLellan et al. found insufficient evidence to determine the optimal fluid strategy for hsPDA, highlighting the need for individualized approaches [94]. Adjustments based on daily fluid balance, weight changes, ventilator settings, and echocardiographic assessment of hemodynamic status are essential to achieving an optimal balance between organ perfusion, respiratory stability, and adequate nutrition. Diuretics may provide temporary respiratory benefits by reducing pulmonary fluid, but there is limited evidence supporting their role in improving long-term outcomes for preterm infants with PDA [95]. We strongly recommend against the routine use of diuretics for PDA management in preterm infants due to limited evidence of long-term benefit and potential for harm (Strength: Strong; Quality of evidence: Low).

### 6.2. Impact of hsPDA on Gastrointestinal Function and Enteral Nutrition (EN)

Hemodynamically significant PDA may impair gastrointestinal function by reducing intestinal blood flow. Normally, enteral feeding increases superior mesenteric artery (SMA) blood flow velocity and splanchnic oxygenation (SrSO_2_) to support digestion [96,97,98,99,100,101]. However, studies on early enteral feeding in VLBW preterm infants showed that those with hsPDA have lower baseline SMA blood flow velocity, and after small-volume feeding, their SMA blood flow and SrSO_2_ remained low without the expected physiological increase. Despite these findings, the differences between preterm infants with and without PDA did not reach statistical significance [102]. These findings suggested that while hsPDA affects intestinal blood flow, its impact on feeding tolerance and NEC or SIP remained unclear. While preterm infants with hsPDA took longer to reach full enteral feeding, this delay did not significantly increase gastrointestinal complications or risk of NEC [102,103]. A study by Martini et al. found that initiating enteral feeding within 72 h of birth, regardless of PDA status, did not affect feeding progression or bowel complications. No significant differences in splanchnic oxygenation or feeding intolerance were observed (Quality of evidence: Low) [104]. These findings suggest that cautious feeding strategies, rather than PDA itself, may contribute to delayed nutrition and a higher risk of malnutrition in preterm infants.

Currently, there is no consensus on the optimal EN strategy for infants receiving PDA medication. A randomized controlled trial by Clyman et al. found no significant difference in NEC or SIP between preterm infants receiving indomethacin or ibuprofen while on trophic feeding (15–60 mL/kg/day) and those who remained fasting. Infants on trophic feeding achieved full EN sooner [105]. Similarly, a retrospective study by Louis et al. reported that continuing trophic feeding during indomethacin treatment shortened the time to full enteral feeding without increasing gastrointestinal complications [106]. We conditionally recommend maintaining trophic feeding during pharmacologic treatment of PDA in preterm infants, as it may be beneficial and does not appear to increase adverse outcomes (Strength: Conditional (weak); Quality of evidence: Low to Moderate).

### 6.3. Summary

There are no standardized guidelines for fluid or EN in preterm infants with hsPDA, and management is often based on clinician experience and individual conditions. While moderate enteral feeding is generally safe and may support better nutritional establishment, concerns about its physiological link to NEC persist. Clinicians should modify feeding strategies based on individual physiological conditions (e.g., IUGR, hypoxia) and clinical status (e.g., hemodynamic stability, use of vasopressors, red blood cell transfusion, type of milk) for comprehensive and personalized care.

## 7. Retinopathy of Prematurity (ROP) and Nutritional Challenges

With the increasing survival rate of extremely preterm infants, the risk of ROP is also rising [107]. Development of ROP is multifactorial and progresses in two phases: An initial phase of oxidative stress, inflammation, and inadequate nutrition leading to impaired vascular growth, followed by either normal vascularization or abnormal neovascularization, which increases the risk of retinal detachment [108,109]. Key risk factors include low gestational age, fluctuating oxygen levels, insufficient weight gain, and low insulin-like growth factor-1 (IGF-1) levels [110]. The abrupt loss of placental nutrition and high oxygen exposure after birth contribute to retinal vessel arrest. Restricted postnatal growth further elevates the risk of ROP [111], emphasizing the importance of optimized nutrition in retinal development. Nutritional management and the care of preterm neonates with ROP are summarized in Figure 1.

### 7.1. Physiological Basis of Nutritional Strategies for ROP Prevention

1. IGF-1 and Early Growth: IGF-1 deficiency and poor early postnatal growth contribute to ROP development.

IGF-1 is crucial for somatic growth and vascular endothelial growth factor (VEGF)-induced retinal vascularization, linking postnatal growth to ROP [112,113]. Preterm birth disrupts the natural rise in IGF-1 during the third trimester, leading to deficiencies that are worsened by infection, oxidative stress, and malnutrition [114]. Since IGF-1 is nutrition-dependent, studies suggested that early postnatal nutrition improves IGF-1 levels and enhances growth, resulting in reduced risk of ROP [111,115,116,117]. Research indicated that low energy intake in the first four weeks increased the risk of severe ROP [116], while early aggressive PN raised IGF-1 levels and consequently lowered the incidence of ROP [118]. Therefore, early nutritional support is vital for preventing ROP in preterm infants (Quality of evidence: Moderate to Low).

2. Optimized Early Nutrition: Adequate energy, lipid, and protein intake support optimal growth and reduce ROP.

Adequate early energy and nutrient intake support postnatal growth and organ maturation in preterm infants. An observational cohort study showed that increasing calorie intake by 10 kcal/kg/day in the first 4 weeks reduced the risk of severe ROP by 24% in ELBW infants (Quality of evidence: Low) [119]. Early high lipid supplementation (≥1.5 g/kg/day within 24 h) also lowered the risk of ROP without safety concerns (Quality of evidence: Moderate) [120,121]. While the role of high amino acid intake in the prevention of ROP remained unclear [111,119], aggressive early protein supplementation is recommended, as IGF-1 is upregulated by increased energy and protein intake [118]. Timely adherence to nutritional guidelines is critical for the prevention of ROP.

3. Hyperglycemia and ROP: Elevated blood glucose levels are linked to an increased incidence of ROP.

Hyperglycemia is a common metabolic issue in preterm infants receiving PN and is linked to an increased risk of ROP [122]. Although the exact mechanism in humans remains unclear, studies in neonatal animal models suggested that hyperglycemia disrupted retinal vascular development by triggering VEGF production and altering photoreceptor metabolism [123,124]. It is also associated with lower serum IGF-1 levels [125]. Meta-analyses studies indicated a tendency for hyperglycemia to increase the risk of ROP [122,126], while research further suggested that both the threshold and duration of hyperglycemia contributed to its development [127]. These findings highlight the importance of glucose regulation in the prevention of ROP.

4. Long-Chain Polyunsaturated Fatty Acids (LCPUFAs) in ROP Prevention: LCPUFAs support retinal development and may lower ROP risk.

Docosahexaenoic Acid (DHA, Omega-3 LCPUFA) and Arachidonic Acid (ARA, Omega-6 LCPUFA) are essential lipid components of membrane phospholipids in the brain and retina [128,129], playing key roles in metabolism, inflammation, and angiogenesis. Preterm birth disrupts the maternal transfer of these LCPUFAs in the third trimester, leading to low reserves and deficiencies that impair retinal development. Since diet is the primary source of DHA and ARA, adequate supplementation is crucial for maintaining an optimal Omega-6 to Omega-3 balance and supporting proper retinal function.

### 7.2. Special Consideration in Enteral Nutrition (EN)

Maternal human milk: Enteral nutrition is crucial for preterm infants, promoting growth, gut microbiome development, and protection against severe ROP [130]. Breast milk is the most effective nutritional intervention for the prevention of ROP due to its bioactive components that support immune modulation, antioxidant activity, and retinal development [131,132,133]. It also provides essential nutrients such as DHA, ARA, and IGF-1 [134]. While its lower calorie and protein content can be addressed through fortification, early enteral nutrition with maternal human milk remains the best strategy to reduce prolonged PN and minimize the risk of ROP.

LCPUFAs supplementation: Studies on sole enteral DHA supplementation showed inconsistent results [135,136], highlighting the importance of ARA [137,138]. A clinical trial by Hellström et al. found that enteral ARA (100 mg/kg/day) and DHA (50 mg/kg/day) in a 2:1 ratio increased serum levels of both fatty acids and reduced the risk of severe ROP by 50% (Quality of evidence: Moderate) [139]. Aligning with these findings, the ESPGHAN guidelines recommend DHA (30–65 mg/kg/day) with ARA (30–100 mg/kg/day) in a ratio of 0.5 to 2 [4]. Balanced enteral supplementation of DHA and ARA is crucial for the prevention of ROP.

Micronutrients: Vitamin E, due to its antioxidant properties, might help reduce the risk of ROP, but high-dose intravenous supplementation increases the risk of sepsis [140,141]. Vitamin A supports retinal development, though its role in the prevention of ROP remains unclear [142,143]. Given the limited evidence, supplementation should follow guidelines: ASPEN recommends 700–1500 IU/kg/day of vitamin A and 6–12 IU/kg/day of vitamin E for enteral use [12], while ESPGHAN advises 1333–3300 IU/kg/day of vitamin A and 2.2–11 mg/kg/day of vitamin E [4]. Zinc, essential for growth and immune function, is lower in preterm infants, and breast milk may not fully meet needs. However, additional enteral zinc supplementation showed no obvious protective effects against ROP [144].

### 7.3. Special Consideration in Parenteral Nutrition (PN)

Risks of Prolonged Parenteral Nutrition: PN is essential for early growth, but prolonged use increases the risk of ROP, possibly due to inadequate nutrient composition, lack of enteral stimulation, and its association with BPD, NEC, and late-onset sepsis [41]. Preterm infants receiving PN for ≥14 days faced an 84% higher risk of ROP and a 120% greater likelihood of requiring treatment (Quality of evidence: Moderate to Low) [145].

Avoidance of hyperglycemia: Insulin is an effective treatment for hyperglycemia, but its impact on the risk of ROP remains controversial due to limited evidence [126,127]. Some studies suggest benefits such as reduced mortality and improved short-term outcomes [146]. On the other hand, the use of insulin carries risks, including hypoglycemia, which is linked to poor neurological outcomes [122]. The ASPEN guidelines advise against routine use of insulin [10], while ESPGHAN guidelines recommend avoiding blood glucose levels above 8 mmol/L (145 mg/dL) and considering insulin therapy only if levels exceed 10 mmol/L (180 mg/dL) despite appropriate glucose infusion management [7]. A safer and more effective strategy to prevent hyperglycemia involves early enteral feeding and adequate protein supplementation, which also supports postnatal growth and reduces the risk of ROP.

LCPUFA supplementation: Fish oil-based intravenous lipid emulsions (rich in omega-3 LCPUFAs) have been linked to a reduced risk of ROP [147], but results remain inconsistent. Some studies show a lower incidence of severe ROP or need for laser therapy [148], while others reported no significant difference compared to non-fish oil emulsions [149]. A meta-analysis study in 2017 suggested potential benefits [147], but a Cochrane review article in 2019 found no clear advantage [150]. Additionally, ELBW infants receiving fish oil emulsions still experienced declining DHA and ARA levels after birth [151], suggesting that current parenteral supplementation may be inadequate.

Micronutrients: Due to limited evidence supporting additional vitamin E and A supplementation, it is recommended to follow international guidelines. Both ASPEN and ESPGHAN guidelines recommend 700–1500 IU/kg/day of vitamin A and 2.8–3.5 IU/kg/day of vitamin E for parenteral administration [12,36].

### 7.4. Summary

Early enteral feeding and adequate protein intake help prevent hyperglycemia. Fish oil-based intravenous lipid emulsions may be beneficial, though the evidence remains inconsistent. We strongly recommend the use of maternal human milk as the primary nutritional strategy to prevent ROP in preterm infants due to the presence of bioactive components such as DHA, ARA, and IGF-1. (Strength: Strong; Quality of evidence: Moderate). We conditionally recommend balanced enteral supplementation of LCPUFAs, including DHA and ARA, in preterm infants at risk of ROP. (Strength: Conditional; Quality of evidence: Low to Moderate).

## 8. Limitations

Limitations in our review exist due to the heterogeneity of diagnostic criteria for specific morbidities across studies conducted in different eras, which may affect the comparability and generalizability of the findings. This review provides general considerations for nutritional strategies in preterm infants under various disease conditions, but it does not offer guidance for personalized nutrition care tailored to the individual needs of each infant—a direction that warrants further research in the future.

## 9. Conclusions

A modified nutritional strategy specialized for preterm infants at risk of morbidities may lead to more favorable outcomes. When feasible, early enteral nutrition combined with the provision of human milk is considered the optimal and safest approach to support appropriate postnatal growth and reduce the incidence of preterm-related complications. However, achieving an optimal nutritional strategy remains a long and challenging journey, as many clinical questions are yet to be resolved, and long-term outcomes, including neurodevelopment and quality of life during childhood, require further investigation.

## Abbreviations

PN, parenteral nutrition; EN, enteral nutrition; HM, human milk; PF, preterm formula; NEC, necrotizing enterocolitis; PMA, postmenstrual age; PDA, persistent ductus arteriosus; MBDP, metabolic bone disease of prematurity; ROP, retinopathy of prematurity; LCPUFAs, long-chain polyunsaturated fatty acids; BPD, bronchopulmonary dysplasia; SIP, spontaneous intestinal perforation.
